# Diagnosis

**Published:** 1861-05

**Authors:** W. H. Atkinson


### DIAGNOSIS.

(Read before the Mississippi Valley Dental Association, Feb. 20, 1861.)

BY DR. W. H. ATKINSON.

DIAGNOSIS, from the Greek words *Δια*, through, and *γινωσκω*, to know. Hence, to see through a case to demonstrative perception of the physical condition present.

One may be accurately acquainted with the exact sciences to the full limit of chemical equivalents and the mutations of numbers, and able correctly to reply to all interrogatories touching anatomy and known physiology and the true theories of pathology, so far as they can be taught by books or words, without specific actual cases to "*seal the instruction*," and yet be unable to diagnose the simplest case of disease presented in actual practice; simply because the knowledge has not become his own by the certain illumination of perceptive unfoldment enabling him to comprehend appearances.

Answers to set questions, although correctly rendered, is no evidence that such answers are anything more than the mere parrotting of a sharp memory of *words*, which, alas! is but too often the method pursued in examinations of pupils in nearly all schools of learning, literary as well as professional; each teacher or professor being more careful to obtain replies in set forms memorised than even better replies in the pupil's own language, which should be preferred, being so much better test of advancement.

To be in *earnest* in our instruction to pupil or patient, or those in the charge of those upon whom we are to exercise our skill, though a prime necessity of doing all the good we can, is by no means the end of our duty as teacher or adviser.

As teacher, our examinations of pupils should be varied and searching in each point, until we are *sure* he does comprehend and make the knowledge his *own.*

As adviser, at the risk even of seeming tedious or over careful, we should require them to rehearse *h w* they understand our instructions, which will always show us that if we had not made this repetition, some points of importance would have escaped them, and thus laid the foundation of failure to fulfill requirements on their part, to the detriment of the cause and our reputation.

Diagnosis, to be useful, must not only be clear and satisfactory to our own minds, but made so plain to those in charge of the case as to make them *alive* to the real necessities present; thus securing that coöperation, without which all our cases will be retarded, if not rendered altogether nugatory, or a real damage to those we had essayed to benefit.

Instructions, ever so minutely given and earnestly enforced, are less efficient for good than when unaccompanied with a clear and philosophical, yet plain statement of the reasons upon which they rest.

I am fully aware that this sort of earnest mode of practice is no less a novelty than the other more laborious methods of performing faithful works.

Easy and superficial methods of investigation are not the most successful roads to certainty of diagnostic competence.

No competent diagnoser will set up his authority as *the* standard, but will always rely upon the perception of those to whom he wishes to make it clear, and in this very democracy (of the most elevated power in reach of the professional man) is there safety, for this very recapitulation of the case may illuminate either his own or the perception of those to whom delineating upon points that had before escaped notice, thus securing certainty in his own mind and the confidence of the other.

It may be asked, who will be the best diagnoser? The answer is very short in its wholeness, and is,—he who knows most.

If, then, the correctness of practice be proportioned to the accuracy of diagnosis, what shall we say of the profession as it now stands? Ans. That it is now doing a great deal of work that will at once become unnecessary and obsolete the moment this one requirement of correct diagnosis becomes more common among us.

Many, very much too many, in the "*practice*," I will not say "*profession*," are *making* more work than they are aware of, for want of this power among men who *are trying* to be honest and live up to the light they have.

One principal reason for this dearth of diagnostic ability had its origin in capable operators employing men to do work in the laboratory for them, by working to models made from the impressions taken by their own hands. After a time, it becomes necessary on some occasions to have this "workman" insert the piece thus made in the mouth of the patient—a few repetitions of which inflated him into a perfect dentist in his own imagination; and upon the slightest pretence, or without assigning reasons at all, he at once moves across the street, and comes out as *the* man to whom you owe your good name as a dentist, and now informs the people that he will do the same sort of services at very reduced rates

By these, and such as these, we have had the field of practice completely filled with (some) honest, ignorant geniuses, who undertake to do alike the doable and the undoable feats of professional skill, in cards that completely exhaust all the living and dead languages, in search of superlatives, and then fail to half lay open to the people their marvelous ability.

It was really marvelous to them and their immediate acquaintances how it was possible for them in so short a time to have attained such eminence. They were astonished at their wonderful productions as really as the boy ever was upon his first successful attempt to construct a whistle in the season when "the bark peeled." And if they *knowing* these things to be so, were thus taken by surprise in finding their cup of mental appreciation full, why, of course, they must needs, like all who have drank but shallow draughts, be intoxicated and run over in mercy to bless a perishing world, as all quacks ever do really in their littleness suppose themselves capable of doing, and in their honesty in telling the world so, expose themselves to be but in their babyhood, which by no means ranks them with the smallest class of grown human beings, so far as bodily proportions go.

And lest we seem too severe on these, let us saddle the right horse, and say at once, those members of the profession are held to answer, who, for favoritism or enormous pupilage fee, have put these Yankee geniuses, possessed of real valuable handicraft, into the profession, telling them, after a four weeks' pupilage, that now they might go and seek their fortunes, for they were much better qualified than they were when they first commenced to do a fine business, if they do not actually give them certificates, as I know some have, stating their competency to be quite equal to the signers as to skill and integrity.

Now, here we have our hands full of good material to make first class dentists of, but they would be insulted if we were to approach them and frankly state to them the patent truth. They are so sure that they know all that is worth knowing, that they can not discern the difference between a well and skillfully made piece in the very height of art, and a piece put up *a la* Tinman's solder, and maintain positively, in the presence of their betters, that the latter was just as *good* as the former specimen.

Now, they must first be convicted of their need of being saved from their error, and then what a rush we should see to the portals of our dental Colleges, *flocking* literally as doves to their windows. If such a movement could be made within a year or two, the impetus that the profession would gain thereby would forever settle the question of respectability as to talent in the various professions; for, to my certain knowledge, there is no body of men, possessed of so much energy, quick perception, mental capacity, physical endurance, manual dexterity, versatility, nonchalance, want of science, and impudent egotism, almost hopelessly mixed up, as that body of men engaged in operating upon the *teeth* in one form or another, all of course claiming to be at least respectably enabled to perform *any* operation they may be called upon for, or *get* it done as well as it can be done by the *best.* "Eternal vigilance" here, as in government, proving "the price of liberty."

And now that excess of liberty has dawned upon all, it becomes those capable, to so regulate it that it proves not destructive. Fire burns equally when inserted in its flame the finger of the innocent and of the hardened offender. So here, also, the good and the erudite are made to suffer and bear a load put upon them by the bad and ignorant, whether they have been "*particeps criminis*" in precipitating this state of things or not.

No doubt we have among us many who are now, through long and severe experiences, quite as competent, after having *learned*, to the cost of their consciences and the well-being of many an innocent victim, how not only to diagnose, but perform a useful service, as those who now honor the profession, without having passed the hells of mal-practice, to arrive at the heaven of "sweet capability" by coming into the profession through the door of a proper education in its schools.

But as it is not every one who says "Lord, Lord, that shall enter into the kingdom," so it is not every one who sports a genuine sheepskin, conferring D. D. S. or M. D., or both combined, that is thus honoring the profession and noble human nature by their practices.

Is it at all questionable whether this ability alone, in its wholeness, would not absolutely annihilate the word fail from our practices?

To my apprehension, complete diagnostic power in faithful exercise would point the certain way to annihilate first one and then another and another abberration from the standard of health, until by the inductive suggestions attained through it, there would be no discordant molecule left to set up the catalysis of disintegrative force.

It is pertinent to ask whether the diagnostic exercise of the mind be a natural gift, or if it is capable of being taught? To which I would reply, that ' all we have we did assuredly receive ;" but if we only keep it in the state in which we received it, there would not be enough in possession of the power to establish a rule to govern us. And, therefore, like our other powers, it must be cultivated, to give us a useful control over it. Like the fire, a wild electricity in the circumambient air, being actively and positively there, but until it has been set in motion by the proper means, and concentrated in the helix or the Leyden jar, it is not in our power to give it direction by our will. But the instant it is so harnessed and brought into harmonious relation with our mental centers by the various means of conducting the current, we may discharge the battery or jar by gentle, unseen induction, by visible sparks, or in one grand, terrific explosion, just as the caprice of the will may dictate.

So every item of knowledge the professional mind has stored away in its Leyden jar serves to intensify any particular mental effort, worthy of the name of effort, in the inductive, spark-like, or explosive form. (I suppose this might explain the immoderate disgust and indignation professional men formerly manifested when any one dared to express a doubt of their skill or integrity—they were so charged with this science motor, as to explode when promptly brought in contact with a powerful conductor, suddenly closing the circuit upon their central battery, self-love.)

The various precious odors owe their value to the capability of being volatilized and scattered when wished to be put to service, and then all within the sphere of influence appreciate and enjoy them. But that they may be transported from place to place for our pleasure or profit, they must be "*sealed*" tightly, and deprived of the liberty to escape. In like manner, all instruction must be "*sealed*" also, to enable the mind to retain and transport it for pleasure or profit. Now, this sealing of instruction is but the closing of the circuit of complete demonstrative teaching by nature or competent leaders in the departments of science.

He who is most nearly harmonised by an equal unfoldment of mental powers in the various departments of his mind will be in possession of most knowledge, and have it also best at command.

Those fragments of education incongruously conjoined, that stand out in angularity, and repel us by their very sharpness, may indicate strength, but are not usefully harmonised until after transposition and retransposition, to train their angles into graceful curves, out of which eventually to produce the beautiful lines that undulate into spherity, the type of complete harmony.

Those best versed in the deeper sciences are usually not the most disposed to communicate freely to others, and often find themselves excluded by self-isolation from what is going on in the new fields of investigation and experiment by their earnestness to confirm or explode the doctrine of their predecessors; whereas, if they would only open themselves to let others partake of their labors, even at the risk of being snubbed by their inferiors, they would soon prove that it is "more blessed to give than to receive," and become possessed of the fresh means of advancement, stript of the worn out nomenclature of the former time.

In our profession especially, do we all need to be "*students*" all our days, and freely discard our most cherished opinions and beliefs, for clear demonstrations of actual knowledge, irrespective of the source from whence it comes.

Lapse of time spent in any calling does not alone confer superiority in execution of its proper function. Neither does long blundering in our specialty of surgery confer the ability to diagnose well. No doubt many a very young member of it may put to shame the veterans, in years, by his superior insight into cases. But other things being equal, length of experience, or in other words, volume of practice and variety does confer this coveted superiority—so that it is not to be expected of beginners to have great, or even considerable skill in examining cases. This, if true, would dictate the propriety, not to say necessity of leaving the eye of long experience, if possible, in every office where patients apply for advice or operations.

The people have heretofore *known* so little of what it was reasonable to expect and require at the hand of the operator that any one with liberal *address* could *get* any amount of work—but, alas! it took the competent dentist to keep it, as soon as they had been instructed by the failures that taught both operator and patient that they had been on the wrong path.

Such an amount of intelligence is now extant, that but a short time comparatively will elapse until the *people* will demand better preparation for the responsible exercise of our humane calling.

The ground properly embraced in the physical structure and diagnosis of human teeth in health and disease has so recently been turned up to the sunlight of scientific observation and cultivation, that it has of necessity given an impetus to collateral science that they will soon have to acknowledge, which, however, we should care less for than to know that it has thus furthered their interests. Surgery and chemistry in particular have been thus benefitted by the researches of Dentistry. How shall the tyro best employ his time to attain the one prime necessity of a successful career in practice?

Not in the examination of cases he never can see again, or more than a limited number of times at irregular periods. Not in hasty, crowded private practice, where no time nor ability, if the patient consented, were allowed to fully delincate the nice distinctions of the various stages of disease in the diverse structures presented. But in such cases as, in whosesoever hands, can be seen from inception to termination, of which the pupil takes sketches and notes in his own language, and reads and discusses in the presence of some one able and *willing* to "point" and "seal" the wholesome instruction to his enlargement, so that he have the type of one class of cases from which he need never to depart. Next, a case nearly allied to this, but with marked modification of temperament, sex, nationality, or other molecular difference, from whatever cause.

And so on through all the modifying circumstances of the entire routine of oral diseases and aberrations from the perfectly healthy standard. If such a course of diagnostic instruction were faithfully followed, we would soon be made aware of the increased ability of our profession in this very important particular, and our pupils would return to their former tutors to turn the tables upon them, and prove to them that if they were younger in years, that they were their seniors in useful ability to see through the various cases daily presented.

To be a good diagnoser it is imperative to understand principles and laws in preference to formularies and modes. And to be able to comprehend these, native powers must not only be good, but kept in action by continued application, thus ensuring the healthy growth so much needed to make their "vigilance" the watch of "intelligence," that strikes down none but foes in the dark or in the light.

That the greatest impetus may be given to diagnostic power, it behooves those who have attained this high ability to so clearly point the way to inquirers, that they may begin where we leave off, which can not be done unless we relate our failures in opposition to our successes, to constitute the sure basis of demonstrative teaching that shall be able to convince the most rigid hair splitter that we meet, by the diffusion of *all* we *know* for the good it may do.

